# Application of Multi-SNP Approaches Bayesian LASSO and AUC-RF to Detect Main Effects of Inflammatory-Gene Variants Associated with Bladder Cancer Risk

**DOI:** 10.1371/journal.pone.0083745

**Published:** 2013-12-31

**Authors:** Evangelina López de Maturana, Yuanqing Ye, M. Luz Calle, Nathaniel Rothman, Víctor Urrea, Manolis Kogevinas, Sandra Petrus, Stephen J. Chanock, Adonina Tardón, Montserrat García-Closas, Anna González-Neira, Gemma Vellalta, Alfredo Carrato, Arcadi Navarro, Belén Lorente-Galdós, Debra T. Silverman, Francisco X. Real, Xifeng Wu, Núria Malats

**Affiliations:** 1 Genetic and Molecular Epidemiology Group, Spanish National Cancer Research Center (CNIO), Madrid, Spain; 2 Department of Epidemiology, The University of Texas MD Anderson Cancer Center, Houston, Texas, United States of America; 3 Systems Biology Department, University of Vic, Vic, Spain; 4 Division of Cancer Epidemiology and Genetics, National Cancer Institute, Department of Health and Human Services, Bethesda, Maryland, United States of America; 5 Centre for Research in Environmental Epidemiology (CREAL), Barcelona, Spain; 6 Institut Municipal d’Investigació Mèdica – Hospital del Mar, Barcelona, Spain; 7 Universidad de Oviedo, Oviedo, Spain; 8 Hospital Universitario de Elche, Elche, Spain; 9 Hospital Universitario Ramon y Cajal, Madrid, Spain; 10 Departament de Ciències Experimentals i de la Salut, Universitat Pompeu Fabra, Barcelona, Spain; 11 Institut de Biologia Evolutiva (UPF-CSIC), Barcelona, Spain; 12 Institució Catalana de Recerca i Estudis Avançats (ICREA), Barcelona, Spain; 13 Instituto Nacional de Bioinformática, Barcelona, Spain; National Taiwan University, Taiwan

## Abstract

The relationship between inflammation and cancer is well established in several tumor types, including bladder cancer. We performed an association study between 886 inflammatory-gene variants and bladder cancer risk in 1,047 cases and 988 controls from the Spanish Bladder Cancer (SBC)/EPICURO Study. A preliminary exploration with the widely used univariate logistic regression approach did not identify any significant SNP after correcting for multiple testing. We further applied two more comprehensive methods to capture the complexity of bladder cancer genetic susceptibility: Bayesian Threshold LASSO (BTL), a regularized regression method, and AUC-Random Forest, a machine-learning algorithm. Both approaches explore the joint effect of markers. BTL analysis identified a signature of 37 SNPs in 34 genes showing an association with bladder cancer. AUC-RF detected an optimal predictive subset of 56 SNPs. 13 SNPs were identified by both methods in the total population. Using resources from the Texas Bladder Cancer study we were able to replicate 30% of the SNPs assessed. The associations between inflammatory SNPs and bladder cancer were reexamined among non-smokers to eliminate the effect of tobacco, one of the strongest and most prevalent environmental risk factor for this tumor. A 9 SNP-signature was detected by BTL. Here we report, for the first time, a set of SNP in inflammatory genes jointly associated with bladder cancer risk. These results highlight the importance of the complex structure of genetic susceptibility associated with cancer risk.

## Introduction

Bladder cancer (BC) is the fifth most common neoplasm in terms of incidence in industrialized countries accounting for approximately 5–7% and 2–2.5% of the newly diagnosed malignancies in men and women, respectively. BC is one of the most prevalent cancers due to its chronic nature [1]. Tobacco and occupational exposure to aromatic amines are the two best established environmental risk factors [2], [3]. In addition, strong evidence for the influence of common genetic variants on BC development has been acquired in the last years [4], [5]. Genetic susceptibility to BC has been investigated in relation to genes encoding enzymes involved in the metabolism of xenobiotics, apoptosis, cell cycle control, angiogenesis, and inflammation [4]. As for the latter process, there is evidence that inflammatory cells, proinflammatory cytokines, and chemokines contribute to immunosuppression, cancer growth, and progression [6]. A link between chronic inflammation and BC is supported by the associations found between *Schistosoma haematobium* and squamous cell carcinoma [7] and, less consistently, between urothelial cell carcinoma and other types of urinary tract infection [8]. In addition, the protective effect of long-term use of non-steroidal anti-inflammatory drugs observed in some case-controls studies supports a role of inflammation in this cancer [9], [10].

Most association studies have focused on the detection of main effects by using an allele- or genotype-based test for each single-nucleotide polymorphism (SNP) separately. However, it is known that complex traits, including BC, are explained by multiple loci with rather small individual effects [11]. Thus, this simple strategy will probably capture only a small proportion of the total genetic variance of the disease conferred by all variants [12]. Therefore, strategies to assess at the same time multiple SNPs and their interaction effects are needed. Standard statistical methods such as logistic regression are not well suited to this end. This level of genetic complexity represents a statistical challenge in association studies because of the high number of regression coefficients (*p*) in comparison to sample size (*n*). Machine learning algorithms provide alternatives for performing multi-SNP analysis [13]. These algorithms are highly appealing since they are model specification-free and may capture hidden information. Random Forest (RF), a classification algorithm proposed by Breiman [14] that can be used to identify the most important variables related to the disease, has also been successfully applied to genome wide data [15]. Recently, an algorithm for variable selection has been proposed (AUC-RF): it identifies the set of variables with the highest predictive accuracy by optimizing the AUC (the area under the ROC curve) of a sequence of random forests [16]. Other methods to deal with oversaturated regression problems [17] that are gaining recognition are the regularized regression methods, such as ridge regression [18], the Least Absolute Shrinkage and Selection Operator (LASSO) [19], and its Bayesian version [20]. These methods are penalized likelihood procedures where suitable penalty functions are added to the negative log-likelihood to automatically shrink spurious effects (effects of redundant covariates) towards zero while effectively estimating the relevant ones. The Bayesian version of LASSO provides several advantages over ridge regression or the classical LASSO. As other Bayesian models, it provides measures of uncertainty about estimates and predictions, and as a consequence, valid standard errors, which can be problematic for the frequentist LASSO [21]. In addition, it yields marker-specific shrinkage of effect estimates, in contrast to ridge regression, and overcomes the main limitation of LASSO which admits at most *n*-1 nonzero regression coefficients [22].

Until present, whole genome association studies (GWAS) individually analyzed a huge number of SNPs, most of them located in regions not associated with the trait of interest while others in LD with the causal variant. This approach is unsatisfactory for traits affected by a large number of variants/genes [12]. An alternative strategy is pathway analysis, dealing with the joint assessment of a subset of SNPs with a potential functional effect on the phenotype of interest.

The main objective of this study was to assess whether SNPs in inflammation-related genes play a role in BC development in a large case control study conducted in Spain and, subsequently, to identify a pattern of those variants (signature) associated with the BC risk by applying two recently developed statistical methods, Bayesian threshold LASSO (BTL) model and AUC-RF. To assess the robustness of the strategy, relevant findings were also analyzed in an independent study, the Texas Bladder Cancer Study.

## Results

### Summary statistics

Table 1 shows the characteristics of cases and controls for the whole sample and for the non-smoker subpopulation. Overall, the study comprised 1,047 cases and 988 controls with genotyping data for 886 SNPs in 194 inflammatory genes. The non-smoker subset consisted of 424 individuals, 147 of which were BC cases. The median age of patients at diagnosis was 68 and 70 years (ranges 22–80 yrs) for the total population and non-smokers, respectively. Overall, cigarette smoking was more common in cases than in controls (86% *vs.* 72%) and in men than in women (87% *vs.* 22%). Consequently, the percentage of men was different in both sets of individuals: 87% and 35% for the total study and for non-smokers, respectively.

**Table 1:** Characteristic profile of the studied population.

	Total population					Non-smoker subset			
	Cases			Controls		Cases		Controls	
	n = 1047		(%)	n = 988	%	n = 147	%	n = 277	%
*Gender*									
Male	915	(87)		873	(88)	52	(35)	180	(65)
Female	132	(13)		115	(12)	95	(65)	97	(35)
*Smoking status*									
Never smokers	147	(14)		277	(28)	147	(100)	277	(100)
Occasional smokers	44	(4)		79	(8)				
Former smokers	400	(38)		361	(37)				
Current smokers	450	(43)		267	(27)				
*Geographical area*									
Barcelona	182	(17)		196	(20)	22	(15)	45	(16)
Vallès/Bages	171	(16)		157	(16)	21	(14)	38	(14)
Elche	77	(7)		79	(8)	15	(10)	27	(10)
Asturias	180	(17)		146	(15)	26	(18)	35	(13)
Tenerife	437	(42)		410	(41)	63	(43)	132	(48)

### Total population analysis

**Figure 1:**
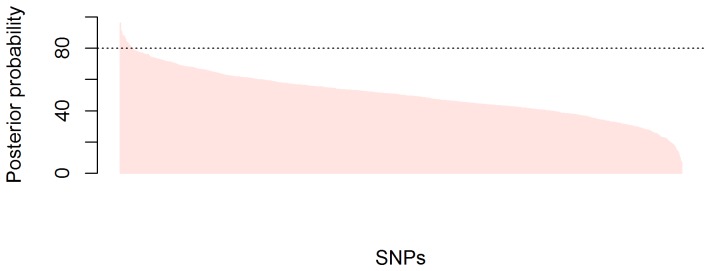
Histogram of the posterior probabilities of having a positive (negative) SNP effect by Bayesian Threshold LASSO model (BTL) in the total population. The dot point line indicates the cut-off point of 80% above which SNPs were considered.

The application of the Bayesian Threshold LASSO provides for each SNP its posterior probability of being associated with BC. In Figure 1, we show the distribution of the posterior probability of each SNP, ranked in decreasing order. SNPs were considered to be associated to BC if the posterior probability of being higher/lower than 0 was > 80%. This strategy identified 37 SNPs in 34 genes showing an association with BC. The highest posterior probability (i.e., most relevant association) was 96.07% for *CASP3-*rs3087455, whereas the lowest one was 51.98% for *TLR2-*rs3804100. The SNPs with a protective minor allele were: *CASP3-*rs3087455, *CCR3-*rs3091312, *CASP9-*rs2020902, *IL17A-*rs8193036, *MAP3K7-*rs150126, *IL6R-*rs8192284, *BLNK-*rs3789928, *SCARB1-*rs4765621, *FOS-*rs7101, *TBK1-*rs10878176, *BIRC5-*rs744120, *LY96-*rs17226566, *AICDA-*rs11046349, *MAP2K4-*rs4791489, *IL15-*rs17461269, *CD14_IK-r*s2569190, *JAK3-*rs11888 and *TNFRSF10A*-rs4871857. The OR posterior means ranged from 0.81 to 0.93 when comparing the minor with the common homozygous genotypes (Table 2). The SNPs with the minor allele associated with an increased risk of BC were: *PRF1-*rs10999426, *IL7R-*rs1494555, *ABCA1-*rs2230806, *IFNAR2-*rs2236757, *MASP1-*rs710459, *BLNK-*rs12357751, *MAP3K3-*rs7209435, *BLNK-*rs10882755, *TLR2-*rs3804099, *SOCS6-*rs723279, *IL17C-*rs899729, *TLR4-rs2737191*, *FOS-*rs1063169, *ABCC4-*rs3765535, *PARP4-*rs13428, *BIRC3-*rs11602147, *IL21R-*rs8049804, *FADD*-rs7939734 and *ICAM1-*rs5498. The posterior means of ORs ranged from 1.10 to 1.20, when comparing the minor with common homozygous genotypes. All the detected SNPs were in Hardy-Weinberg equilibrium in the control population. Single-SNP logistic regression models yielded *p-values* <0.05 for 17 of them (of a total of 32, see Table S1) with a minimum *p*-*value* of 0.0021, not corrected by multiple testing. The estimated OR corresponding to the 37 SNPs-signature was >4.92 (see Figures S1 and S2 for more details). The 95% interval for the OR when comparing the highest risk genotype combination with the highest protective one ranged from 31.2–629.4. The wide range of the credibility interval shows the large error associated with the estimate. Posterior mean, median and mode of the asymmetric posterior distribution were 206.5, 123.5 and 63.8, respectively.

**Table 2:** Risk estimates of Bayesian Threshold LASSO model (BTL), considering a posterior probability higher than 80%, and from logistic regression for the total population.

SNP	Gene	Type	Position	Alleles	OR_aa_AA_ a	Post prob^b^	OR_aa_AA_ c	*p*-value^d^
**rs3087455**	***CASP3***	**Intronic**	**4q35.1**	**A/C**	**0.81**	**96.07**	**0.66**	**0.002**
**rs10999426**	***PRF1***	**Intronic**	**10q22.1**	**A/G**	**1.20**	**93.33**	**1.36**	**0.025**
**rs1494555**	***IL7R***	**non_synonymous coding**	**5p13.2**	**G/A**	**1.19**	**92.30**	**1.43**	**0.015**
**rs2230806**	***ABCA1***	**non_synonymous coding**	**9q31.1**	**G/A**	**1.19**	**92.05**	**1.38**	**0.029**
rs3091312	*CCR3*	downstream	3p21.31	A/T	0.86	90.93	0.69	0.013
**rs8192284**	***IL6R***	**non_synonymous coding**	**1q21.3**	**A/G**	**0.88**	**90.35**	**0.74**	**0.022**
rs2236757	*IFNAR2*	intronic	21q22.11	A/G	1.16	89.77	1.43	0.015
rs8193036	*IL17A*	upstream	6p12.2	C/T	0.88	88.50	0.74	0.048
**rs710459**	***MASP1***	**intronic**	**3q27.3**	**C/T**	**1.14**	**88.17**	**1.23**	**0.113**
**rs4765621**	***SCARB1***	**intronic**	**12q24.31**	**G/A**	**0.89**	**87.88**	**0.78**	**0.063**
rs150126	*MAP3K7*	intronic	6q15	G/A	0.88	87.74	0.68	0.011
rs3789928	*BLNK*	intronic	10q24.1	G/C	0.88	87.63	0.78	0.069
rs7209435	*MAP3K3*	intronic	17q23.3	T/C	1.13	86.29	1.29	0.095
rs2020902	*CASP9*	splice site	1p36.21	T/C	0.88	86.19	0.61	0.007
**rs899729**	***IL17C***	**upstream**	**16q24.3**	**C/A**	**1.12**	**86.06**	**1.22**	**0.132**
rs7101	*FOS*	5’ UTR	14q24.3	C/T	0.89	85.93	0.71	0.023
rs12357751	*BLNK*	intronic	10q24.1	C/T	1.14	85.67	1.36	0.040
**rs3804099**	***TLR2***	**synonymous coding**	**4q31.1**	**T/C**	**1.13**	**84.97**	**1.27**	**0.072**
rs2737191	*TLR4*	upstream	9q33.1	A/G	1.12	84.53	1.28	0.088
**rs4791489**	***MAP2K4***	**downstream**	**17p12**	**C/T**	**0.91**	**84.52**	**0.80**	**0.112**
rs10878176	*TBK1*	intronic	12q14.2	G/C	0.90	83.88	0.70	0.014
rs17226566	*LY96*	intronic	8q21.11	T/C	0.90	83.47	0.77	0.108
rs744120	*BIRC5*	upstream	17q25.3	C/G	0.90	83.27	0.69	0.014
rs723279	*SOCS6*	intronic	18q22.2	G/A	1.12	83.16	1.28	0.097
rs13428	*PARP4*	**non_synonymous coding**	13q12.12	G/C	1.11	83.11	1.26	0.094
rs11046349	*AICDA*	3’ UTR	12p13.31	T/G	0.91	82.17	0.60	0.014
rs11602147	*BIRC3*	intronic	11q22.2	C/G	1.10	81.98	1.23	0.148
rs1063169	*FOS*	intronic	14q24.3	G/T	1.12	81.88	1.56	0.018
**rs2569190**	***CD14_IK***	**5’ UTR**	**5q31.3**	**A/G**	**0.92**	**81.83**	**0.75**	**0.025**
**rs5498**	***ICAM1***	**coding unknown**	**19q13.2**	**HGMD mutation**	**1.10**	**81.24**	**1.25**	**0.087**
rs17461269	*IL15*	intronic	4q31.21	T/A	0.91	81.22	0.76	0.072
rs3765535	*ABCC4*	intronic	13q32.1	A/G	1.12	81.21	1.41	0.091
rs11888	*JAK3*	3’ UTR	19p13.11	T/C	0.92	81.15	0.84	0.212
rs10882755	*BLNK*	intronic	10q24.1	A/G	1.13	80.76	1.41	0.058
**rs7939734**	***FADD***	**upstream**	**11q13.3**	**T/A**	**1.10**	**80.75**	**1.22**	**0.135**
rs8049804	*IL21R*	intergenic	17q22	A/C	1.10	80.33	1.18	0.239
rs4871857	*TNFRSF10A*	non synonymous coding	8p21.3	G/C	0.93	80.22	0.80	0.082

^a^ Posterior mean of the OR, calculated from the BTL analyses Similar values for the median were obtained for each SNP.

^b^ It corresponds to 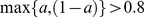, where 
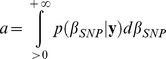
.

^c^ OR obtained from the adjusted logistic regression.

^d^ *p*-value of the trend obtained from the adjusted logistic regression.

SNPs also selected by AUC-RF are bold-faced.

AUC-RF considered both genetic and non-genetic variables and detected an optimal subset of 59 factors, including 56 SNPs (Table S2). All the environmental covariates, except for gender, were ranked first: smoking status was ranked as the most relevant variable, with a Mean Decrease Gini index (MDG) of 11.55, followed by geographical region with a relative importance of 35.2%. The age of the patient was ranked in the third place with a relative importance of 19.4%, followed by SNPs. Table 3 shows the 12 most important SNPs detected by this method. Their relative importance ranged from 20.8% for *JAK3*-rs2286662 to 14.4% for *AKR1C3*-rs1937845.

**Table 3:** Relative variable importance for the top 12 polymorphisms selected by AUC-RF in the total population.

rs number	Gene	Type	Alleles	Position	Relative variable importance a
rs2286662	*JAK3*	non synonymous coding	A/G	19p13.11	20.8%
rs8192284	*IL6R*	non_synonymous coding	A/C	1q21.3	16.4%
rs7104333	*CD5*	downstream	A/G	11q12.2	16.3%
rs288980	*ROCK1*	intronic	C/T	18q11.2	15.7%
rs3087455	*CASP3*	intronic	A/C	4q35.1	15.6%
rs11655650	*BIRC5*	intronic	C/T	17q25.3	15.6%
rs3213427	*CD4*	3’ UTR	T/C	12p13.31	15.3%
rs3136701	*CD2*	intronic	C/G	1p13.1	15.0%
rs4765621	*SCARB1*	intronic	G/A	12q24.31	14.8%
rs5498	*ICAM1*	coding unknown	A/G	19p13.2	14.8%
rs2839488	*TFF1*	intronic	C/G	21q22.3	14.6%
rs1937845	*AKR1C3*	5’ UTR	G/A	10p15.1	14.4%

a Calculated by dividing the raw variable importance measurement by that with the highest MDG, that of smoking status.

**Figure 2:**
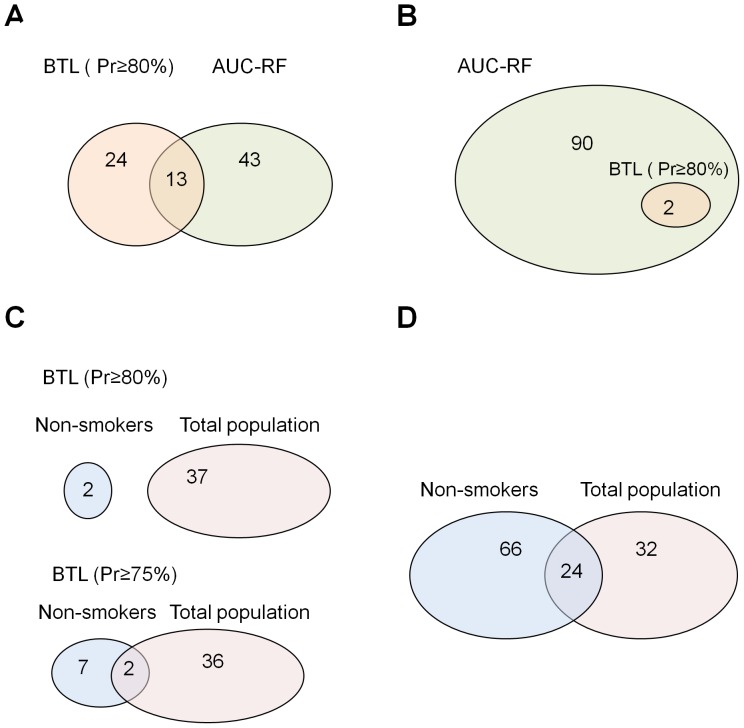
Venn diagrams showing the overlapping between the SNPs selected by Bayesian Threshold model (BTL) and AUC-Random Forest (AUC-RF). (A) Number of SNPs detected by each method in the total population. (B) Number of SNPs detected by each method in the non-smoker subset. (C) Number of common SNPs detected by BTL in the total population and non-smoker subset, with posterior probabilities of at least 80% and 75% of having an effect different from 0. (D) Number of SNPs detected by AUC-RF in both the total population and the non-smoker subset.

Thirteen SNPs in *CASP3*, *PRF1*, *IL7R*, *ABCA1*, *IL6R*, *MASP1*, *SCARB1*, *TLR2*, *IL17C*, *MAP2K4*, *CD14_IK*, *FADD*, and *ICAM1* were identified as relevant by both BTL and AUC-RF approaches (bold-faced SNPs in Table 2; see also Figure 2a.). Among them, 6 SNPs located in *CASP3*, *PRF1*, *IL7R*, *ABCA1*, *IL6R* and *CD14_IK* had a *p-value*<0.05 by logistic regression adjusted by covariates (see Table 2, for more details). The significance of none of them held after Bonferroni correction for multiple testing [23]. Despite the fact that no significant association was found after performing the single marker analyses, the ranking of SNPs highly correlated with that obtained from the posterior probability BTL-based results (Spearman’s correlation, rho =  0.78).

Genotypes for 17/37 SNPs with a posterior probability higher than 80% in the discovery phase were available from the TXBC study and this information was used for replication purposes. In addition, 13 SNPs in high LD with SNPs detected by BTL in the discovery phase were included in the phase 2 analyses. Table S3 shows the posterior probabilities of being larger/smaller than 0 and the posterior mean of ORs obtained in the replication set. Two SNPs (*IL6R-*rs4129267 and *TBK1-*rs10878182) in high LD with *IL6R*-rs8192284 and *TBK1-*rs10878176 detected in the discovery study by BTL had posterior probabilities of having a non-null effect higher than 90%. The OR of these surrogate SNPs were of risk while those identified in the discovery study were of protection. Five additional SNPs (*IL21R-*rs9930086 - in high LD with *IL21R*-rs8049804, and *MAP3K3-*rs7209435, IL17A-rs8193036, FADD-rs7939734, and TLR2-rs3804099) showed posterior probabilities >70%, the threshold considered for replication. The ORs of these 5 SNPs were of the same magnitude and direction as those found in the discovery study.

### Non-smoker subset analysis

Tobacco smoking is the strongest and most prevalent environmental risk factor for BC and it may modify the effect of SNPs in inflammation-related genes. Therefore, we performed the association analysis among non-smokers to bypass its effect. In such a context, BTL detected only two relevant SNPs (*BCL10-*2647396 and *NFKBIA-*rs696) associated with the risk of BC with a posterior probability of at least 80%. The two SNPs were also detected by AUC-RF (see Figure 2b). When we extended the posterior probability (≥75%), the number of SNPs detected by both approaches increased up to 8 in 8 genes (see Table 4). OR posterior means ranged from 1.12–1.16 for those SNPs showing an increased risk of BC, when comparing the two homozygous genotypes, and from 0.89–0.91 for those with a protective effect. Univariate logistic regression analysis yielded significant results for the 8 SNPs with a minimum *p*-*value* of 0.0032, not corrected by multiple testing. The OR median posterior density corresponding to the 9 SNPs-signature detected by BTL was 2.73, with a posterior probability of 99% of being >1 and a range between 1.35 and 6.66 as 95% credible interval (see Figure S3).

**Table 4:** Risk estimates from Bayesian Threshold LASSO model (BTL), considering a posterior probability of 75%, and from logistic regression analyses among non-smokers.

rs number	Gene	Type	Position	Alleles	OR_aa_AA_ a	Post prob^b^	OR_aa_AA_ c	*p*-value^d^
**rs696**	***NFKBIA***	**3’ UTR**	**14q13.2**	**A/G**	**1.16**	**79.97**	**2.47**	**0.004**
**rs2647396**	***BCL10***	**intronic**	**1p22.3**	**C/T**	**0.89**	**79.65**	**0.40**	**0.011**
**rs10999426**	***PRF1***	**intronic**	**10q22.1**	**A/G**	**1.15**	**78.67**	**2.11**	**0.019**
**rs1800890**	***IL10***	**upstream**	**1q32.1**	**A/T**	**1.13**	**76.48**	**2.26**	**0.019**
**rs812606**	***MAP3K7***	**intronic**	**6q15**	**C/T**	**1.13**	**76.37**	**2.68**	**0.005**
**rs4791489**	***MAP2K4***	**downstream**	**17p12**	**C/T**	**0.91**	**76.87**	**0.49**	**0.030**
rs20432	*PTGS2*	intronic	1q31.1	T/G	0.91	75.17	0.28	0.003
**rs11188660**	***BLNK***	**intronic**	**10q24.1**	**C/T**	**1.12**	**75.66**	**2.56**	**0.004**
**rs1061217**	***SLAMF1***	**3’ UTR**	**1q23.3**	**T/C**	**1.12**	**75.89**	**1.73**	**0.077**

a Posterior mean of the OR, calculated from the BTL analyses. Similar values for the median were obtained for each SNP.

b It corresponds to 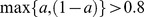, where 
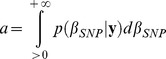
.

c OR obtained from the adjusted logistic regression.

d *p*-value of the trend obtained from the adjusted logistic regression.

SNPs also selected by AUC-RF are bold-faced.

AUC-RF detected an optimal subset of 93 variables related to BC, 90 of which were SNPs (Table S4). Contrary to the findings in the total population, gender was the most important covariate related to BC among non-smokers, and age and region were at the third and fourth position, respectively.

### Common SNPs between total and non-smoker datasets

Figures 2c and 2d show the number of SNPs detected by both BTL and AUC-RF in the SBC/EPICURO study for both the whole population and the non-smoker individuals. There were no common SNPs detected by BTL for those population sets with posterior probabilities greater than 80%. However, when the posterior probability applied was ≥75%, three SNPs were detected in both datasets: *MAP2K4-*rs4791489, *PRF1*-rs10999426 and *BCL10*-rs2647396.

When focusing on AUC-RF results, 24 SNPs (*ABCA1-*rs2230806, *AICDA-*rs2580874, *ALOX5-*rs1369214, *BCL10-*rs2647396, *CD2-*rs3136701, *CD4-*rs2707210, *FADD-*rs7939734, *FASLG-*rs929087, *H2AFX-*rs640603, *H2AFX-*rs643788, *IKBKB-*rs3747811, *IL15RA-*rs2296135, *IL21R-*rs2189521, *JAK3-*rs2286662, *MAP2K4-*rs4791489, *MASP1-*rs710459, *NFKBIA-*rs696, *OPRD1-*rs204076, *PRF1-*rs10999426, *RELA-*rs11820062, *RELA-*rs1466462, *SCARB1-*rs4765621, *TBK1*-rs10878178 and *TMED7-*rs2052834) were identified in both datasets, representing the 43% and 27% of the SNPs selected in total and non-smoker subjects, respectively.

## Discussion

As all complex diseases, BC is not a single SNP/gene disorder. Rather, many SNPs with small effects may lead to the impairment of key pathways involved in their pathophysiology. The identification of such SNP-signatures represents an analytical challenge requiring the application of novel comprehensive statistical approaches. To our knowledge, this is the first study on BC analyzing a large number of SNPs with BTL that has identified a subset of them jointly contributing to this phenotype with a relevant magnitude of risk, much higher than that provided by smoking (OR = 5 [2]), the main risk factor for BC.

Thirteen SNPs in 13 genes were identified by both BTL and AUC-RF, which can be considered as an internal validation. SNPs in *CASP3*, *IL6R*, *SCARB1*, *MAP2K4* and *CD14_IK* showed a protective effect whereas those in *PRF1*, *IL17R*, *ABCA1*, *MASP1*, *TLR2*, *IL17C*, *FADD* and *ICAM1* were associated with a higher risk of BC. Each SNP showed a small individual effect that could not have been identified by logistic regression, the common analytical approach used in GWAS, after applying the conservative Bonferroni’s correction for multiple testing.

We found previously published evidences about the association of several of these SNPs/ genes with cancer risk despite the fact that this information was not used in SNP selection. Among them, *SCARB1* codes for the scavenger receptor class B type I gene, a cell-surface receptor that binds to high-density lipoprotein cholesterol (HDL-C) and mediates HDL-C uptake [24], [25]. *SCARB1*-rs4765621 maps to intron 1 and has been associated with an increased risk of BC in combination with *SLC23A2*-rs12479919, *AKR1C3*-rs2275928 and *PLA2G6-*rs2016755 [26]. This SNP is in linkage disequilibrium with *SCARB1*-rs4765623 that has been associated with renal cell carcinoma [27]. *MAP2K4* encodes a dual specificity Ser/Thr protein kinase. Allelic imbalances in this gene have been reported in bladder tumors [28]. Furthermore, deletions and mutations of the *MAP2K4* have been described in human pancreatic, lung, breast, testicle, and colorectal cancer cell lines, suggesting a tumor suppressor role [29]. *MAP2K4-*rs4791489 is located 1226 bp downstream of the gene and this is the first study to report an association with a phenotype. *IL7R* encodes the receptor for IL-7, a cytokine involved in the T cell differentiation and activation. *IL7R* variation has been linked to chronic inflammatory diseases and cancer: *IL7R*-rs1494555 has been associated with an increased risk of gastric cancer [30], hematological neoplasms - by interacting with a high BMI - [31], and non-small cell lung cancer where it was detected by both logistic regression and random forest tests [31]. This SNP leads to a Ile^138^Val substitution for which there is no functional evidence. *CD14* plays a major role in pathogen-activated signal transduction pathways and in the production of inflammatory cytokines [32]. *CD14_IK-*rs2569190 has been associated with prostate cancer in African Americans [33],and with coronary artery and cerebrovascular diseases [34], [35]. ***PRF1*** codes for perforin 1, one of the main toxic proteins of cytolytic granules and a key effector in T-cell- and natural killer-cell-mediated cytolysis. Its alterations cause familial hemophagocytic lymphohistiocytosis type 2 (HPLH2), a rare and lethal autosomal recessive disorder of early childhood. *PRF1*-rs10999426 has been clustered with other genes associated with cytotoxic T cells in a colorectal cancer study: high expression of the cytotoxic cluster genes was associated with a prolonged disease-free survival [36]. Soluble interleukin-6-receptor-α-subunit (*IL-6R*) is a potent cytokine playing an important role in immune response. Altered gene expression has been associated with multiple myeloma, autoimmune diseases and prostate cancer risk [37]. The SNP *IL6R*-rs7529229, in linkage disequilibrium with *IL6R*-rs8192284, has also been related to risk of multiple myeloma [37].

We further focused on the assessment of non-smokers to discard the potential modifying effect of tobacco on the association between genetic variants and bladder cancer risk. Only two polymorphisms associated with BC were detected by both analytical methods: *NFKBIA-rs696* and *BCL10-rs2647396*. *NFKBI* is involved in response to stress, regulates *COX-2* and proinflammatory cytokines, and is an important mediator of oncogenesis [38]. The *NFKBIA-rs696* homozygosity has been associated with a poorer survival in Swedish patients with colorectal cancer [39]. Other studies have associated the deletion of *NFKBIA* with glioblastoma multiforme [40] and Hodgkin’s lymphoma specimens [41]. *NFKBIA-*rs696 is in linkage disequilibrium with rs8904, a variant that has been associated with pain severity in lung cancer patients [42]. *BCL10*, associated with protection from BC in our study, plays an important role in the NF-kappaB and STAT signalling pathways [40], It has been proposed to participate in pancreatic carcinoma [43] and *MALT* lymphomas as part of the t(1,4)(p22, q32) translocation [44]. *BCL10-rs2647396* is intronic and no function is known for this polymorphism.

Using an independent population and surrogate SNPs in high LD with those identified in the discovery study, we replicated the association with SNPs in *IL6R* and *TBK1* identified by BTL. The fact that the ORs obtained in the replication study were in opposite direction to those detected in the discovery study can be explained when using surrogate SNPs. Greene *et al.* recently proved with simulated data that differences in allele frequency can also provide an inversed allelic effect in a replication study [45]. When the threshold of the posterior probability was lowered to 70%, the association of five additional SNPs was also replicated. Overall, we were able to replicate 30% of the selected SNPs by BTL available in the TXBC study, a figure that is remarkable when considering that BC is largely caused by environmental factors and that both studies come from different geographical areas and from centers with distinct patient referral patterns (in the SBCS study most centers are general hospitals whereas the TXBC Study was conducted at MD Anderson Cancer Center). Other proposed causes for lack of replication are genetic heterogeneity, environmental interactions, age-dependent effects, inadequate statistical power, and gene-gene interactions, the latter explanation pointing to a higher complexity of the underlying genetic architecture [45]. We did not attempt to replicate SNPs identified by AUC-RF because this method depends largely on the initial variables considered. Woefully, data from a number of the original SNPs considered in the discovery phase were not available in the study used for replication.

The present study has several major strengths. Importantly, it applies innovative analytical approaches dealing with the biological complexity of the phenotype. Association analyses were carried out by applying a regularized regression model (BTL) and a nonparametric variable selection method (AUC-RF), in addition to the single marker unconditional logistic regression, used in most association studies. The first two methods overcome the main limitation of the latter since they consider all the genetic information jointly. The application of individual logistic regression makes sense under the assumption that only few genes affect genetic predisposition [12], which certainly is not the case for BC. BTL considers, a priori, that most of the SNPs have a small (if any) effect on disease development, and performs a marker specific shrinkage of effect estimates [20]. This approach permits dealing with the “small *n* large *p”* problem and prevents overfitting. De los Campos et al [22] suggested this method as an interesting alternative to perform regressions on markers under an additive model. We considered as associated to BC those SNPs with a posterior probability >0.8 of having an effect greater (smaller) than 0, as in [45]. Other criteria, as the Bayesian LOD score >3.2 [46] or “heritability of the marker”>0.5% [47], have been used in previous applications of BL. The choice of these criteria is arbitrary because they have not been formally compared yet. On the contrary, AUC-RF does not assume any model and considers all possible interactions between the covariates included in the analyses. It provides a measure of the importance of the variable, although it does not indicate whether the effect of this variable is protective or risky. It is also important to emphasize that the selected variables with AUC-RF are not necessarily significantly associated with the trait; rather, they represent the combination of genotypes that best predicts the disease indicator and are thus worthy of further investigation. We gave priority to those SNPs selected by both methods although SNPs selected by only one of them should not be discarded, given the different nature and assumptions of each approach. Further methodological strengths of the study are the large sample size, the high participation rates, and the high quality of information on exposures and genotyping of the SBC/EPICURO Study.

However, some limitations need to be considered when interpreting these results. It is possible that potentially informative susceptibility markers were not selected for genotyping. In addition, incomplete tagging of the selected genes may have resulted from the use of an earlier HapMap release to select tag SNPs. Therefore, those genes with SNPs without relevant results in this study should not be disregarded as potentially associated with BC. As for the constraints of the approaches used, BTL only assumes an additive mode of inheritance and no interactions were considered. A common drawback of machine learning based methods, such as AUC-RF, is that they typically identify a SNP set that produces the highest classification accuracy but does not necessarily correspond to a strong association with the disease. Indeed, machine learning-based approaches tend to introduce false positives, since the inclusion of many SNPs increases classification accuracies [48].

The large difference in the risk estimates according to BTL between the total and the non-smoker datasets suggests a potential modifying effect of tobacco over the SNP-signature on BC risk. While statistical underpowered results cannot be discarded, a large smoking*SNPs interaction assessment considering all SNPs included in the study should be performed. This analysis requires of further methodology innovation and large computational infrastructure.

In conclusion, we report here the joint effect of several variants in inflammatory genes strongly associated with BC risk. The use of multi-SNP assessment approaches to explore the hidden heritability of complex diseases is highly promising in the association analysis field. While the application of these methods at a genome-wide level is straightforward, the great computational demand represents the main constraint and few studies have applied them to genome-wide data in association [15] or prediction settings [49] till present. Ours is one of the first studies applying such methodologies to a large set of SNPs in cancer research.

## Materials and Methods

### Ethics statement

Informed written consent was obtained from the study participants. The study was approved by the Institutional Review Board of the U.S. National Cancer Institute, the Ethics Committees of each participating hospital, MD Anderson Cancer Center, and the Baylor College of Medicine.

### Study population

The population considered in this analysis comes from the Spanish Bladder Cancer/EPICURO Study. This is a hospital-based case-control study conducted during 1998–2001 in 18 hospitals in five areas in Spain (Asturias, Barcelona metropolitan area, Vallès/Bages, Alicante and Tenerife), as described elsewhere [50]. Eligible cases were aged 21–80 years and newly diagnosed of a histologically confirmed transitional cell carcinoma of the urinary bladder based on the 1998 system of WHO and the International Society of Urological Pathology [51]. Controls were selected from patients admitted to participating hospitals for diagnoses thought to be unrelated to the BC risk factors, and individually matched to the cases on age at interview within 5-year categories, gender, ethnicity and region. A total of 1,451 cases and 1,444 controls were eligible for the study, out of them 84% of the cases and 88% of the controls agreed to participate and were interviewed.

Demographic and risk factor information was collected at the hospital using computer-assisted personal interviews. Subjects were categorized regarding smoking status as never smokers, if they had smoked less than 100 cigarettes in their lifetime; occasional smokers, if they had smoked at least one cigarette per day for less than 6 months; former smokers, if they had smoked regularly but stopped smoking more than one year before the study inclusion date; and current smokers, if they had smoked regularly within a year of the inclusion date.

A very large proportion of individuals, 1,188 cases and 1,173 controls, provided blood or buccal cell sample for DNA extraction.

### Gene & SNP selection and genotyping

A 1,536 SNPs GoldenGate Illumina Genotyping Assay (San Diego, CA, USA) platform was designed using tagSNPs selected to cover the variation of key genes involved in candidate pathways, among them those related to inflammatory response (see Table S5). Genes were carefully selected according to current available evidence, both epidemiological and biological, of their involvement in inflammatory processes. Gene selection favored those inflammatory genes showing association with cancer. The selection of tagSNPs was done using the SYSNPs (Select Your SNPs) application [52]. At the moment of tagSNPs selection, the available databases were dbSNP b25, hg17 and HapMap Release. The tagger algorithm was Haploview's Tagger (v3.32) with default parameter values. SYSNPs options were selected to force the inclusion of SNPs that had been previously genotyped in the same samples as tagSNPs. These previous genotyping efforts are described in detail in Garcia-Closas [50], [53] for the TaqMan and a previous GoldenGate Illumina genotyping, respectively.

SNPs were genotyped following the manufacturer’s instructions (http://www.illumina.com/) regarding DNA amount and concentrations and by including intra- and inter-assay duplicates as well as negative controls. DNA from cases and controls was blindly placed in the 96-well plates so that proportionate numbers were achieved in all plates. Successful genotyping was obtained for 1,150 cases and 1,149 controls.

### Quality control

Following the recommendations of Anderson, Pettersson et al. [54], a thorough assessment of data quality was carried out. SNPs (N = 15) genotyped in the present and previous platforms showed good agreement (ranging from 98.2% to 99.8%); thus, individuals with missing SNPs data in one platform were completed with their counterparts from the same SNP genotyped in the other platforms, if they were no missing. To consider individuals in this analysis, we required that >95% of the SNPs were successfully genotyped in each platform. In addition, SNPs showing >5% missing genotypes, and those with a MAF <5% that did not show an association with BC after performing a Fisher’s exact test, were excluded from the analyses. The final number of cases and controls included in the analysis was 1,047 and 988, respectively. Missing genotypes were imputed with the *k-*NN method using the package SCRIME in R [55]. To minimize colinearity between variables, pairwise linkage disequilibrium between SNPs was estimated based on *r^2^* values using the package GENETICS in R (http://cran.r-project.org/web/packages/genetics/index.html), and only one of each pair of SNPs were retained for further analyses when *r^2^* >0.8. In addition, those SNPs pertaining to the *X* chromosome (24 SNPs belonging to 7 genes) were excluded from the final dataset, yielding a final number of 886 SNPs and 194 genes. The existence of population stratification was also checked using a principal component analysis. While participants were selected from different regions of Spain, no subpopulations were apparent after the visual inspection of the first two principal components.

### Statistical methods

Three different statistical approaches were applied to explore the association of SNP variants with BC risk: the Bayesian Threshold Lasso model (BTL), the Area Under the Curve-Random Forest (AUC-RF), and the logistic regression applied to each individual SNP.

**Bayesian Threshold LASSO model.** A BTL model [45] was adapted in this study for the association analysis. A threshold model [56] was considered to account for the discrete nature of the phenotype (*y*), which can take the values 0 or 1, if the individual did not show or showed the disease, respectively. This methodology assumes that the expression of the trait is related to an underlying continuous variable, called liability (*l*) [57], so that the disease is observed if the value of the latent variable exceeds a threshold (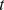). This can be expressed as follows:



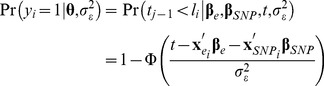
(1)


where 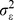 corresponds to the residual variance, 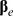 is the vector of environmental (non-genetic) covariate effects (*i. e.*, gender; smoking status in 4 categories; region, in 5 categories; and age as a continuous covariate), 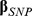 is the vector of the coefficients of *p* SNPs in inflammation related genes, and 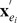 and 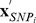 correspond to the incidence vectors of appropriate order. Thus, the following model was assumed for the liabilities 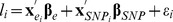. An additive inheritance model was assumed for the SNPs, so that they were coded as 0, 1 and 2 if the genotype was AA, Aa and aa, respectively.

LASSO method [19] simultaneously perform variable selection and shrinkage of coefficients. The Bayesian interpretation of the LASSO [20] indicates that the solution can be viewed as the posterior mode in a Bayesian model with Gaussian likelihood 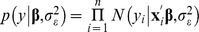 and a prior on 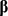 that is the product of *m* independent, zero-mean, double exponential (DE) densities 
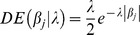
. DE prior can be represented as a mixture of scaled Gaussian densities, where the mixing process of the variances is an exponential distribution (
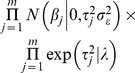
). Parameter 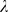 controls the shape of the prior distribution assigned to 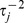, the exponential prior assigns more density to small values of the 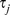 than to large ones, and follows, a priori, a Gamma distribution 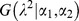. A DE prior produces stronger shrinkage of regression coefficients close to 0 (without an effect) and less shrinkage to those with large absolute values.

Fully conditional distribution for unknowns (i.e., regression coefficients (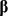), 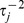
and 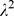) were multivariate normal with mean (covariance matrix) equal to the solution (inverse of coefficient matrix) of the system of equations 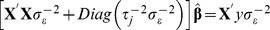, inverse Gaussian with mean 
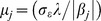
 and scale parameter 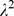, and 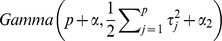, respectively. The residual variance and the threshold separating the two categories (controls and cases) were set to 1 and 0 for identification purposes.

For each analysis, a unique McMC chain of 50,000 iterations was obtained using a Gibbs sampler implemented in the Fortran-language [45]. The first 10,000 iterations were discarded as burn-in and all the remaining iterations were retained to infer posterior marginal distributions of unknown parameters. Convergence of chains was assessed visually and applying the Geweke criterion [58]. SNPs were considered to be associated with BC when the mass of the marginal posterior distribution of the coefficient was far from the null hypothesis being equal to zero, specifically, when 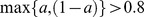, where *a* is the area from zero to infinity under the marginal posterior distribution of the coefficient: 
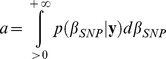
.

Posterior distributions of odds ratios (OR) for each SNP were calculated as a measure of relative risk. First, the probability of having BC for each genotype at each iteration was calculated by transforming the liability to BC to the observable scale, following Dempster and Lerner [59]. Thus, the probability of having BC for individuals with genotype AA for the SNP *s* at iteration (*k*) was calculated as:



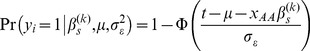
(2)


where 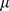 corresponds to the mean of the liability to BC. OR posterior distributions for each SNP were calculated considering the AA genotype as the reference group. For example, the odds of disease for individuals with the heterozygous genotype compared with those individuals with the AA genotype (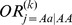) at iteration *k* was calculated as:



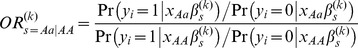
(3)


In addition, the OR posterior distributions corresponding to the signature of the SNPs detected by BTL were calculated using a modification of equation (3):



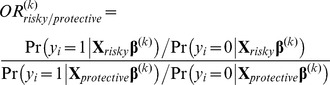
(4)


where 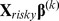/ 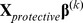 corresponds to the effect of the most risky/protective genotypes of the SNPs detected as relevant by BTL at iteration *k*.

**Area Under the Curve-Random Forest.** Recently Calle et al. [16] proposed a new algorithm for genomic profiling based on optimizing the area-under-the ROC curve (AUC) of the Random Forest [13]. Briefly, RF comprises an ensemble of trees, generated from bootstrap samples, and their application provides a quantification of the variable importance in the context of a non-linear model. AUC-RF algorithm iteratively fits RF and performs a backward elimination process based on the initial ranking of variables. The area under the ROC curve (AUC) of the RF is used instead of the classification error as a measure of predictive accuracy. Five hundred trees were used to construct each forest, a 20% of variables were eliminated at every step and the number of remaining variables to stop the process of backward elimination was set to 10. The optimizing criterion used to construct the trees was the mean decrease Gini index (MDG), and the relative variable importance of each variable was calculated by dividing the raw variable importance measurement (i.e., the MDG) by that with the highest MDG. In order to perform a more fair comparison with the results obtained from BTL model, environmental effects were also included in the AUC-RF analyses. Age, recorded as a continuous variable, was categorized in 3 groups: <60 yr; 60–70 yrs; and ≥ 70 yrs, to avoid bias [60]. AUC-RF was implemented using the AUCRF package [16] in R- language (http://www.R-project.org).

**Logistic regression.** The standard approach in most GWAS was also used to analyze the association between BC and the individual SNPs belonging to genes related to inflammation. Odds ratios and *p*-values for the common homozygous versus the rare homozygous were calculated for each SNP using an unconditional logistic regression. Environmental covariates (e. g., age, region, smoking status and gender) were included in the model in order to adjust by their potential confounding effects. Analyses were run in R-language (http://www.R-project.org). Those SNPs with *p*-values <0.05, two-sided test, after Bonferroni’s correction were kept for comparison with the results of the other two methods.

The number of SNPs detected by both BTL and AUC-RF in the total population and the non-smoker subset was examined, as well as the corresponding *p*-value obtained after the logistic regression. Analyses were also run among non-smokers to clean the population up for the smoking effect, the main environmental risk factor. It has been previously suggested that the genetic architecture of this group is different from that among smokers [16]. Those SNPs detected by the two first methods both in the total population and in the non-smokers subset were further discussed.

### Replication study

Cases and controls in the replication stage were from the Texas Bladder Cancer (TXBC) study, a hospital-based bladder cancer case–control study conducted at the University of Texas MD Anderson Cancer Center. All cases were newly diagnosed, histologically confirmed, and previously untreated incident bladder cancer cases recruited from 1999 until present [5]. The controls were healthy individuals with no prior history of cancer (except non-melanoma skin cancer) and were recruited from Kelsey Seybold, the largest multi-specialty, managed-care physician group in the Houston metropolitan area.

Trained MD Anderson interviewers collected comprehensive epidemiological data in a 45 minute interview on demographics, family history of cancer, and smoking status from all study participants of the TXBC study. Most (78.2%) of cases and sex-matched controls were men. The mean age of cases (63.8 years) and controls (62.9 years) was highly similar. As expected, the percentage of smokers among cases was higher than that among control: 73% vs. 55%, respectively. Blood sample was collected for DNA extraction at the end of the interview.

Genomic DNA extraction and storage was described elsewhere [5]. Genotyping for the TXBC data set was generated using the Illumina HumanHap610 chip containing 620,901 markers and the iSelect chip containing 9,645 SNPs at MD Anderson. Detailed quality control measures were described previously. All the subjects included in this study were Caucasians and had call rate >95%. A total number of 695 cases and 706 controls were considered for analysis. SNP call rate >95% criterion was applied. We further removed markers that deviated from Hardy-Weinberg equilibrium in the controls at *p*-*value* < 0.0001. Out of 886 SNPs considered in the discovery study, 372 SNPs were available from the TXBC study and passed quality control procedures. Among them, 17 SNPs overlapped with those selected by the BTL method in the SBC/EPICURO Study. We further included 13 SNPs from the TXBC study that were in high LD with SNPs detected in the discovery study. The BTL model included all SNPs as well as gender, age, and smoking status. The analyses were performed with the same software and criteria in both studies.

## Supporting Information

Figure S1 **Posterior distribution of the OR corresponding to the 37 SNP-signature detected by BTL in the total population (calculated using equation 4).** The minimum estimate for OR was 4.92. The 95% credible interval for the ORs corresponding to the 37 SNPs-signature ranged 31.15-629.42 with the most risky genotype combination conferring 123.5 (posterior median) fold higher risk of BC than the most protective one. The wide 95% interval indicates a large error associated to the estimation of the OR for the 37 SNPs-signature.
(DOCX)

Figure S2 **Log OR median (bullets) and 95% credible intervals (vertical lines) for the signatures including the SNPs with the highest posterior probabilities from BTL analysis on total data.** Note that the higher number of SNPs included in the signature the wider is the 95% credible interval (error associated to the estimate of OR).
(DOCX)

Figure S3 **Posterior distribution of the OR corresponding to the 9 SNP-signature detected by BTL (75% posterior probability cut-off point) in the non-smoker subset.** Note that the 95% credible interval is narrower than that for the OR of the SNP-signature for the total population (Figures S1 and S2), probably due to the lower number of SNPs included in that signature.
(DOCX)

Table S1 **Genotype distribution among cases and controls and bladder cancer risk estimates for the SNPs with** ***p*** **-values <0.05 obtained in the single marker analyses.**
(DOCX)

Table S2 **Relative variable importance for the polymorphisms selected by AUC-RF in the total population.**
(DOCX)

Table S3 **Risk estimates and posterior probabilities of having an effect larger (smaller) than 0 of the SNPs included in the validation study that are in common (or in high LD) with those detected in the discovery study by BTL.**
(DOCX)

Table S4 **Relative variable importance for the SNPs selected by AUC-RF among the non-smoker population.**
(DOCX)

Table S5 **Top 50 gene sets of canonical pathways and GO biological process (** http://www.broadinstitute.org/gsea **) with the rate of overlapping with the gene set used in this study.**
(DOCX)
