# Supplementary material for: Application of Multi-SNP Approaches Bayesian LASSO and AUC-RF to Detect Main Effects of Inflammatory-Gene Variants Associated with Bladder Cancer Risk
Source: PLoS One. 2013 Dec 31;8(12):e83745. doi: 10.1371/journal.pone.0083745 (PMC3877090; doi:10.1371/journal.pone.0083745)
Supplement: Table S1 — Genotype distribution among cases and controls and bladder cancer risk estimates for the SNPs with p -values <0.05 obtained in the single marker analyses. (DOCX) [file pone.0083745.s004.docx]

Table S1

|  |  | Controls | | | Cases | | |  |  |
| --- | --- | --- | --- | --- | --- | --- | --- | --- | --- |
| SNP | Gene | AA | Aa | aa | AA | Aa | aa | ORaa_AA | *p*-value |
| rs3087455 | *CASP3* | 440 | 421 | 127 | 531 | 402 | 114 | 0.66 | 0.002 |
| rs2020902 | *CASP9* | 695 | 263 | 30 | 791 | 233 | 23 | 0.61 | 0.007 |
| rs150126 | *MAP3K7* | 557 | 363 | 68 | 650 | 340 | 57 | 0.68 | 0.011 |
| rs3091312 | *CCR3* | 524 | 388 | 76 | 607 | 386 | 54 | 0.69 | 0.013 |
| rs10878176 | *TBK1* | 484 | 411 | 93 | 561 | 408 | 78 | 0.70 | 0.014 |
| rs744120 | *BIRC5* | 524 | 387 | 77 | 603 | 391 | 53 | 0.69 | 0.014 |
| rs11046349 | *AICDA* | 750 | 229 | 9 | 842 | 194 | 11 | 0.60 | 0.014 |
| rs2236757 | *IFNAR2* | 553 | 374 | 61 | 543 | 405 | 99 | 1.43 | 0.015 |
| rs1494555 | *IL7R* | 512 | 400 | 76 | 488 | 465 | 94 | 1.43 | 0.015 |
| rs11655650 | *BIRC5* | 353 | 456 | 179 | 404 | 502 | 141 | 0.73 | 0.017 |
| rs1063169 | *FOS* | 755 | 213 | 20 | 755 | 267 | 25 | 1.56 | 0.018 |
| rs4073 | *IL8* | 297 | 486 | 205 | 352 | 502 | 193 | 0.74 | 0.021 |
| rs8192284 | *IL6R* | 316 | 480 | 192 | 392 | 476 | 179 | 0.74 | 0.022 |
| rs11899004 | *CASP8* | 695 | 263 | 30 | 768 | 258 | 21 | 0.66 | 0.022 |
| rs7101 | *FOS* | 515 | 398 | 75 | 595 | 386 | 66 | 0.71 | 0.023 |
| rs10999426 | *PRF1* | 423 | 450 | 115 | 408 | 492 | 147 | 1.36 | 0.025 |
| rs2569190 | *CD14_IK* | 247 | 481 | 260 | 286 | 526 | 235 | 0.75 | 0.025 |
| rs16938758 | *LY96* | 606 | 333 | 49 | 680 | 326 | 41 | 0.70 | 0.029 |
| rs1926188 | *FAS* | 478 | 413 | 97 | 557 | 397 | 93 | 0.73 | 0.029 |
| rs2230806 | *ABCA1* | 528 | 400 | 60 | 515 | 440 | 92 | 1.38 | 0.029 |
| rs16829984 | *CD80* | 766 | 204 | 18 | 844 | 189 | 14 | 0.64 | 0.030 |
| rs4149579 | *TNFRSF1A* | 863 | 119 | 6 | 885 | 152 | 10 | 1.72 | 0.031 |
| rs2272732 | *IKBKB* | 785 | 188 | 15 | 782 | 251 | 14 | 1.54 | 0.034 |
| rs3806798 | *IL15* | 775 | 205 | 8 | 788 | 239 | 20 | 1.52 | 0.039 |
| rs588019 | *CCR7* | 867 | 118 | 3 | 951 | 91 | 5 | 0.56 | 0.039 |
| rs12357751 | *BLNK* | 557 | 370 | 61 | 546 | 418 | 83 | 1.36 | 0.040 |
| rs3756561 | *CD180* | 840 | 145 | 3 | 922 | 118 | 7 | 0.59 | 0.040 |
| rs7101916 | *RELA* | 715 | 253 | 20 | 786 | 249 | 12 | 0.67 | 0.041 |
| rs12722588 | *IL2RA* | 663 | 281 | 44 | 745 | 266 | 36 | 0.71 | 0.041 |
| rs2114169 | *LY96* | 620 | 329 | 39 | 695 | 326 | 26 | 0.71 | 0.045 |
| rs982764 | *FAS* | 532 | 384 | 72 | 523 | 444 | 80 | 1.35 | 0.045 |
| rs8193036 | *IL17A* | 580 | 338 | 70 | 638 | 365 | 44 | 0.74 | 0.048 |
