# Supplementary material for: Application of Multi-SNP Approaches Bayesian LASSO and AUC-RF to Detect Main Effects of Inflammatory-Gene Variants Associated with Bladder Cancer Risk
Source: PLoS One. 2013 Dec 31;8(12):e83745. doi: 10.1371/journal.pone.0083745 (PMC3877090; doi:10.1371/journal.pone.0083745)
Supplement: Table S2 — Relative variable importance for the polymorphisms selected by AUC-RF in the total population. (DOCX) [file pone.0083745.s005.docx]

**Table S2**

| rs number | Gene | Type | Alleles | Position | Relative variable importance ^a^ |
| --- | --- | --- | --- | --- | --- |
| rs2286662 | *JAK3* | non_synonymous coding | A/G | 19p13.11 | 20.8% |
| rs8192284 | *IL6R* | non_synonymous coding | A/C | 1q21.3 | 16.4% |
| rs7104333 | *CD5* | downstream | A/G | 11q12.2 | 16.3% |
| rs288980 | *ROCK1* | intronic | C/T | 18q11.2 | 15.7% |
| rs3087455 | *CASP3* | intronic | A/C | 4q35.1 | 15.6% |
| rs11655650 | *BIRC5* | intronic | C/T | 17q25.3 | 15.6% |
| rs3213427 | *CD4* | 3’ UTR | T/C | 12p13.31 | 15.3% |
| rs3136701 | *CD2* | intronic | C/G | 1p13.1 | 15.0% |
| rs4765621 | *SCARB1* | intronic | G/A | 12q24.31 | 14.8% |
| rs5498 | *ICAM1* | coding unknown | A/G | 19p13.2 | 14.8% |
| rs2839488 | *TFF1* | intronic | C/G | 21q22.3 | 14.6% |
| rs1937845 | *AKR1C3* | 5’ UTR | G/A | 10p15.1 | 14.4% |
| rs2569190 | *CD14_IK* | 5’ UTR | T/C | 5q31.1 | 14.31% |
| rs1369214 | *ALOX5* | intronic | G/A | 10q11.2 | 14.28% |
| rs710459 | *MASP1* | intronic | C/T | 3q27.3 | 14.18% |
| rs4807650 | *TICAM1* | intronic | C/T | 19P13.3 | 14.12% |
| rs944722 | *NOS2A* | intronic | G/A | 17q11.2-q12 | 14.10% |
| rs10878178 | *TBK1* | intronic | C/T | 12q14.2 | 14.06% |
| rs10999426 | *PRF1* | intronic | G/A | 10q22.1 | 14.02% |
| rs11820062 | *RELA* | 5’ UTR | T/C | 11q13.1 | 14.02% |
| rs2025345 | *IL2RA* | intronic | A/G | 10p15.1 | 13.91% |
| rs3804099 | *TLR2* | synonymous | T/C | 4q32 | 13.90% |
| rs3764651 | *ABCA7* | intronic | A/G | 19p13.3 | 13.87% |
| rs2230806 | *ABCA1* | missense | G/A | 9q31.1 | 13.86% |
| rs12693932 | *CASP8* | intronic | T/C | 2q33.1 | 13.85% |
| rs7939734 | *FADD* | upstream | T/A | 11q13.3 | 13.84% |
| rs5030411 | *TRAF6* | intronic | G/A | 11p12 | 13.78% |
| rs11571316 | *CTLA4* | upstream | A/G | 2q33 | 13.78% |
| rs640603 | *H2AFX* | downstream | G/A | 11q23.3 | 13.74% |
| rs899729 | *IL17C* | upstream | C/A | 16q24.3 | 13.70% |
| rs1466462 | *RELA* | downstream | G/C | 11q13.1 | 13.69% |
| rs4073 | *IL8* | upstream | A/T | 4q13.3 | 13.69% |
| rs10898847 | *FADD* | upstream | G/A | 11q13.3 | 13.69% |
| rs1594 | *CFLAR* | synonymous | A/G | 2q33.1 | 13.68% |
| rs2189521 | *IL21R* | 5’ UTR | C/T | 16p12.1 | 13.68% |
| rs696 | *NFKBIA* | 3’ UTR | C/T | 14q13.2 | 13.66% |
| rs643788 | *H2AFX* | missense | T/C | 11q23.3 | 13.65% |
| rs9395767 | *IL17A* | upstream | T/A | 6p12.2 | 13.65% |
| rs929087 | *FASLG* | intronic | A/G | 1q23 | 13.65% |
| rs2296135 | *IL15RA* | 3’ UTR | A/C | 10p15.1 | 13.61% |
| rs3769825 | *CASP8* | intronic | A/G | 2q33.1 | 13.59% |
| rs2707210 | *CD4* | intronic | G/T | 12p13.31 | 13.54% |
| rs1900300 | *MAP3K7IP2* | intronic | T/C | 6q25.1 | 13.52% |
| rs1494555 | *IL7R* | missense | G/A | 5p13.2 | 13.52% |
| rs776514 | *IRAK2* | intronic | T/C | 3p25.3 | 13.51% |
| rs2052834 | *TMED7* | intronic | G/A | 5q22.3 | 13.47% |
| rs1539096 | *PARP4* | synonymous | G/A | 13q12.12 | 13.45% |
| rs2075267 | ARHGDIB | upstream | C/A | 12p12.3 | 13.45% |
| rs3747811 | *IKBKB* | 5’ UTR | A/G/T | 8p11.21 | 13.44% |
| rs4791489 | *MAP2K4* | downstream | C/T | 17p12 | 13.44% |
| rs10931934 | *CASP8* | intronic | T/C | 2q33.1 | 13.44% |
| rs7648642 | *CD80* | intronic | A/C | 3q13.33 | 13.41% |
| rs2580874 | *AICDA* | intronic | G/A | 12p13.31 | 13.41% |
| rs1926188 | *FAS* | downstream | T/C | 10q23.31 | 13.40% |
| rs2647396 | BCL10 | intronic | C/T | 1p22.3 | 13.40% |
| rs204076 | OPRD1 | upstream | T/A | 1p35.3 | 13.40% |
