# Supplementary material for: Application of Multi-SNP Approaches Bayesian LASSO and AUC-RF to Detect Main Effects of Inflammatory-Gene Variants Associated with Bladder Cancer Risk
Source: PLoS One. 2013 Dec 31;8(12):e83745. doi: 10.1371/journal.pone.0083745 (PMC3877090; doi:10.1371/journal.pone.0083745)
Supplement: Table S3 — Risk estimates and posterior probabilities of having an effect larger (smaller) than 0 of the SNPs included in the validation study that are in common (or in high LD) with those detected in the discovery study by BTL. (DOCX) [file pone.0083745.s006.docx]

**Table S3**

| SNPs TXBCS | SNP EPICURO | LD | Gene | OR | Post prob^a^ |
| --- | --- | --- | --- | --- | --- |
| rs4129267 | **rs8192284** | 0.97 | *IL6R* | 1.36 | 0.91 |
| rs10878182 | rs10878176 | 0.96 | *TBK1* | 1.40 | 0.91 |
| rs9930086 | rs8049804 | 1 | *IL21R* | 1.20 | 0.78 |
| rs7209435 |  |  | *MAP3K3* | 1.20 | 0.78 |
| rs8193036 |  |  | *IL17A* | 0.90 | 0.72 |
| **rs7939734** |  |  | *FADD* | 1.13 | 0.71 |
| **rs3804099** |  |  | *TLR2* | 1.13 | 0.71 |
| rs744120 |  |  | *BIRC5* | 1.10 | 0.66 |
| **rs899729** |  |  | *IL17C* | 0.95 | 0.64 |
| rs2020902 |  |  | *CASP9* | 0.94 | 0.64 |
| rs3758562 | **rs10999426** | 0.96 | *PRF1* | 0.94 | 0.64 |
| rs2846848 | rs11602147 | 0.93 | *BIRC3* | 0.95 | 0.64 |
| rs3765535 |  |  | *ABCC4* | 1.11 | 0.63 |
| rs1063169 |  |  | *FOS* | 0.95 | 0.63 |
| rs4765623 | **rs4765621** | 1 | *SCARB1* | 0.96 | 0.62 |
| rs11888 |  |  | *JAK3* | 1.07 | 0.61 |
| rs7101 |  |  | *FOS* | 1.07 | 0.60 |
| rs778584 | **rs2569190** | 1 | *CD14_IK* | 1.06 | 0.60 |
| rs10882754 | rs10882755 | 1 | *BLNK* | 1.08 | 0.60 |
| rs150125 | rs150126 | 1 | *MAP3K7* | 1.06 | 0.60 |
| **rs1494555** |  |  | *IL7R* | 1.06 | 0.59 |
| **rs5498** |  |  | *ICAM1* | 0.97 | 0.59 |
| rs6557634 | rs4871857 | 1 | *TNFRSF10A* | 1.05 | 0.58 |
| rs1050112 | rs13428 | 1 | *PARP4* | 0.98 | 0.58 |
| **rs2230806** |  |  | *ABCA1* | 0.98 | 0.57 |
| rs2236758 | rs2236757 | 0.90 | *IFNAR2* | 1.05 | 0.57 |
| rs11046349 |  |  | *AICDA* | 1.07 | 0.57 |
| rs723279 |  |  | *SOCS6* | 1.03 | 0.54 |
| rs2737191 |  |  | *TLR4* | 1.03 | 0.53 |
| rs7645716 | rs3091312 | 1 | *CCR3* | 1.01 | 0.51 |

SNPs also selected by AUC-RF in EPICURO study are bold-faced

^a^ It corresponds to
