# Supplementary material for: Application of Multi-SNP Approaches Bayesian LASSO and AUC-RF to Detect Main Effects of Inflammatory-Gene Variants Associated with Bladder Cancer Risk
Source: PLoS One. 2013 Dec 31;8(12):e83745. doi: 10.1371/journal.pone.0083745 (PMC3877090; doi:10.1371/journal.pone.0083745)
Supplement: Table S4 — Relative variable importance for the SNPs selected by AUC-RF among the non-smoker population. (DOCX) [file pone.0083745.s007.docx]

**Table S4**

| rs number | Gene | Type | Alleles | Position | Relative variable importance^a^ |
| --- | --- | --- | --- | --- | --- |
| rs11820062 | *RELA* | 5’UTR | T/C | q13.1 | 45.30 |
| rs1466462 | *RELA* | downstream | G/C | q13.1 | 38.91 |
| rs626364 | *CD80* | intronic | G/A | q13.33 | 28.55 |
| rs4490816 | *WWP1* | intronic | T/C | q21.3 | 27.97 |
| rs696 | *NFKBIA* | 3’UTR | C/T | q31.2 | 27.74 |
| rs2230806 | *abca1* | non synonymous coding | C/T | q31.1 | 26.73 |
| rs2189521 | *IL21R* | upstream | C/T | q12.1 | 25.71 |
| rs10999426 | *PRF1* | intronic | G/A | q22.1 | 25.07 |
| rs723279 | *SOCS6* | intronic | G/A | q22.2 | 24.99 |
| rs640603 | *H2AFX* | downstream | G/A | q23.3 | 24.00 |
| rs1369214 | *alox5* | intronic | G/A | q11.21 | 23.93 |
| rs812606 | *MAP3K7* | intronic | A/G | q15 | 23.53 |
| rs8103315 | *BCL3* | intronic | C/A | q13.32 | 22.62 |
| rs2707210 | *CD4* | intronic | G/T | p13.31 | 22.52 |
| rs1801274 | *FCGR2A* | non synonymous coding | A/G | q23.3 | 22.30 |
| rs2647396 | *BCL10* | intronic | C/T | p22.3 | 22.04 |
| rs1061217 | *slamf1* | 3’UTR | A/G | q23.3 | 21.72 |
| rs1800890 | *il10* | upstream | A/T | q32.1 | 21.49 |
| rs643788 | *H2AFX* | non synonymous coding | T/C | q23.3 | 21.44 |
| rs10498658 | *RIPK1* | intronic | C/T | q25.2 | 21.32 |
| rs8078439 | *MAP2K4* | intronic | T/C | p12 | 21.15 |
| rs1144159 | *MAP3K7* | intronic | A/G | q15 | 20.50 |
| rs9642913 | *WWP1* | intronic | G/A | q21.3 | 20.48 |
| rs1786704 | *TIRAP* | intronic | T/C | q24.2 | 19.68 |
| rs4951523 | *TRAF5* | intronic | T/C | q32.3 | 19.64 |
| rs17505589 | *IL7* | intronic | C/T | q21.12 | 19.57 |
| rs8832 | *il4r* | 3’UTR | A/G | p12.1 | 19.39 |
| rs11188660 | *BLNK* | intronic | G/A | q24.1 | 19.26 |
| rs6893629 | *CD180* | upstream | G/C | q12.3 | 19.24 |
| rs962409 | *BCL10* | 3’UTR | A/C | p22.3 | 18.87 |
| rs2580874 | *AICDA* | intronic | G/A | q13.31 | 18.79 |
| rs2052834 | *TMED7* | intronic | G/A | q22.3 | 18.65 |
| rs1059369 | *gdf15* | non synonymous coding | T/A | p13.11 | 18.49 |
| rs2296135 | *il15ra* | 3’UTR | A/C | p15.1 | 18.44 |
| rs1329423 | *cfh* | intronic | T/C | q31.3 | 18.13 |
| rs11465853 | *IRAK2* | intronic | G/C | p25.3 | 17.97 |
| rs1169670 | *IRAK2* | intronic | G/C | p25.3 | 17.74 |
| rs867185 | *nbs1* | intronic | G/A | q21.3 | 17.74 |
| rs11591959 | *BLNK* | intronic | A/G | q24.1 | 17.48 |
| rs4791489 | *MAP2K4* | downstream | C/T | p12 | 17.42 |
| rs3802814 | *TIRAP* | synonymous coding | G/A | q24.2 | 17.36 |
| rs9658786 | *FAS* | downstream | C/T | q23.31 | 17.35 |
| rs3802604 | *GATA3* | intronic | G/A | p14 | 17.34 |
| rs4833095 | *TLR1* | non synonymous coding | T/C | p14 | 17.22 |
| rs2020902 | *CASP9* | splice site | A/G | p36.21 | 17.20 |
| rs2850166 | *AIRE* | intronic | T/C | q22.3 | 17.13 |
| rs1905045 | *LY96* | upstream | C/T | q21.11 | 17.11 |
| rs7373858 | *IRAK2* | intronic | G/A | p25.3 | 17.10 |
| rs8057464 | *IL21R* | intronic | A/C | p12.1 | 17.09 |
| rs2069762 | *il2* | upstream | A/C | q27 | 17.02 |
| rs2286662 | *JAK3* | non synonymous coding | T/C | p13.11 | 16.96 |
| rs10878178 | *TBK1* | intronic | C/T | q14.2 | 16.91 |
| rs713129 | *SOCS6* | intronic | G/C | q22.2 | 16.83 |
| rs10491070 | *BLNK* | intronic | T/C | q24.1 | 16.50 |
| rs12524576 | *ULBP2* | upstream | G/A | q25.1 | 16.45 |
| rs9610 | *il10ra* | 3’UTR | G/A | q23.3 | 16.43 |
| rs6741642 | *SOCS5* | intronic | C/T | p21 | 16.40 |
| rs157682 | *MAP3K7* | intronic | G/A | q15 | 16.40 |
| rs4711998 | *IL17A* | upstream | A/G | p12.2 | 16.39 |
| rs3136701 | *CD2* | intronic | G/C | p13.1 | 16.36 |
| rs1244186 | *GATA3* | within non coding gene | T/C | p14 | 16.25 |
| rs3829223 | *TOLLIP* | intronic | C/T | p15.5 | 16.22 |
| rs1031101 | *mbl2* | upstream | A/G | q21.1 | 16.20 |
| rs929087 | *faslg* | intronic | A/G | q24.3 | 16.19 |
| rs4765621 | *scarb1* | intronic | C/T | q24.31 | 16.19 |
| rs7939734 | *FADD* | upstream | T/A | q13.3 | 16.08 |
| rs2031229 | *IL2RA* | intronic | G/A | 15.1 | 16.05 |
| rs3138056 | *NFKBIA* | downstream | C/T | q13.2 | 16.00 |
| rs5085 | *apoa2* | intronic | C/G | q23.3 | 15.90 |
| rs689466 | *ptgs2* | upstream | T/C | q31.1 | 15.87 |
| rs2525053 | *HDAC7A* | intronic | T/C | q13.11 | 15.85 |
| rs4849091 | *SLC20A1* | synonymous coding | G/A | q13 | 15.84 |
| rs3736149 | *ICBR* | downstream | G/A | q22.3 | 15.83 |
| rs4939364 | *MS4A1* | intronic | A/G | q12.2 | 15.83 |
| rs11602147 | *BIRC3* | intronic | C/G | q22.2 | 15.81 |
| rs12357751 | *BLNK* | intronic | C/T | q24.1 | 15.79 |
| rs7602 | *lepr* | 3’UTR | G/A | p31.3 | 15.78 |
| rs7311222 | *UBE2N* | intronic | A/G | q22 | 15.71 |
| rs710459 | *masp1* | intronic | G/A | q27.3 | 15.71 |
| rs11674814 | *CASP8* | essential splice site | large deletion | q33.1 | 15.70 |
| rs204076 | *oprd1* | downstream | T/A | p35.3 | 15.70 |
| rs2254514 | *il15* | 5’UTR | T/C | q31.21 | 15.58 |
| rs1474326 | *BCL6* | intronic | C/A | q27.3 | 15.52 |
| rs3747811 | *IKBKB* | 5’UTR | A/G/T | p11.21 | 15.49 |
| rs10800309 | *FCGR2A* | upstream | A/G | q23.3 | 15.48 |
| rs17686001 | *MAP3K14* | intronic | G/A | q21.31 | 15.48 |
| rs1137101 | *lepr* | non synonymous coding | A/G | p31.3 | 15.43 |
| rs982764 | *FAS* | intronic | T/C | q23.31 | 15.42 |
| rs3859503 | *NLRP12* | downstream | A/C | q13.42 | 15.42 |
| rs4560769 | *IKBKB* | intronic | A/G | p11.21 | 15.27 |

^a^ Calculated by dividing the MGI of each SNP by the highest one obtained, that for gender.
