# Supplementary material for: Application of Multi-SNP Approaches Bayesian LASSO and AUC-RF to Detect Main Effects of Inflammatory-Gene Variants Associated with Bladder Cancer Risk
Source: PLoS One. 2013 Dec 31;8(12):e83745. doi: 10.1371/journal.pone.0083745 (PMC3877090; doi:10.1371/journal.pone.0083745)
Supplement: Table S5 — Top 50 gene sets of canonical pathways and GO biological process ( http://www.broadinstitute.org/gsea ) with the rate of overlapping with the gene set used in this study. (DOCX) [file pone.0083745.s008.docx]

Table S5

| Gene set name | Description | Overlapping |
| --- | --- | --- |
| BIOCARTA_NFKB_PATHWAY | NF-kB Signaling Pathway | 0.83 |
| BIOCARTA_RELA_PATHWAY | Acetylation and Deacetylation of RelA in The Nucleus | 0.75 |
| BIOCARTA_DC_PATHWAY | Dendritic cells in regulating TH1 and TH2 Development | 0.73 |
| BIOCARTA_TOLL_PATHWAY | Toll-Like Receptor Pathway | 0.68 |
| BIOCARTA_CYTOKINE_PATHWAY | Cytokine Network | 0.67 |
| REACTOME_ACTIVATED_TLR4_SIGNALLING | Genes involved in Activated TLR4 signalling | 0.67 |
| BIOCARTA_TNFR2_PATHWAY | TNFR2 Signaling Pathway | 0.67 |
| REACTOME_TOLL_LIKE_RECEPTOR_4_CASCADE | Genes involved in Toll Like Receptor 4 (TLR4) Cascade | 0.64 |
| ST_TUMOR_NECROSIS_FACTOR_PATHWAY | Tumor Necrosis Factor Pathway. | 0.64 |
| BIOCARTA_STRESS_PATHWAY | TNF/Stress Related Signaling | 0.64 |
| BIOCARTA_TH1TH2_PATHWAY | Th1/Th2 Differentiation | 0.63 |
| REACTOME_MYD88_CASCADE | Genes involved in MyD88 cascade | 0.63 |
| BIOCARTA_NTHI_PATHWAY | NFkB activation by Nontypeable Hemophilus influenzae | 0.63 |
| BIOCARTA_INFLAM_PATHWAY | Cytokines and Inflammatory Response | 0.59 |
| BIOCARTA_IL1R_PATHWAY | Signal transduction through IL1R | 0.58 |
| BIOCARTA_NKT_PATHWAY | Selective expression of chemokine receptors during T-cell polarization | 0.50 |
| BIOCARTA_DEATH_PATHWAY | Induction of apoptosis through DR3 and DR4/5 Death Receptors | 0.48 |
| KEGG_TOLL_LIKE_RECEPTOR_SIGNALING_PATHWAY | Toll-like receptor signaling pathway | 0.41 |
| KEGG_LEISHMANIA_INFECTION | Leishmania infection | 0.38 |
| BIOCARTA_HIVNEF_PATHWAY | HIV-I Nef: negative effector of Fas and TNF | 0.36 |
| REACTOME_TOLL_RECEPTOR_CASCADES | Genes involved in Toll Receptor Cascades | 0.34 |
| KEGG_APOPTOSIS | Apoptosis | 0.33 |
| I_KAPPAB_KINASE_NF_KAPPAB_CASCADE | Genes annotated by the GO term GO:0007249. A series of reactions initiated by the activation of the transcription factor NF-kappaB. NF-kappaB is sequestered by the inhibitor I-kappaB, and is released when I-kappaB is phosphorylated by activated I-kappaB kinase. | 0.33 |
| POSITIVE_REGULATION_OF_I_KAPPAB_KINASE_NF_KAPPAB_CASCADE | Genes annotated by the GO term GO:0043123. Any process that activates or increases the frequency, rate or extent of an I-kappaB kinase/NF-kappaB induced cascade. | 0.31 |
| KEGG_NOD_LIKE_RECEPTOR_SIGNALING_PATHWAY | NOD-like receptor signaling pathway | 0.31 |
| REGULATION_OF_I_KAPPAB_KINASE_NF_KAPPAB_CASCADE | Genes annotated by the GO term GO:0043122. Any process that modulates an I-kappaB kinase/NF-kappaB induced cascade. | 0.30 |
| CYTOKINE_PRODUCTION | Genes annotated by the GO term GO:0001816. The appearance of a cytokine due to biosynthesis or secretion following a cellular stimulus, resulting in an increase in its intracellular or extracellular levels. | 0.29 |
| KEGG_RIG_I_LIKE_RECEPTOR_SIGNALING_PATHWAY | RIG-I-like receptor signaling pathway | 0.28 |
| POSITIVE_REGULATION_OF_CELLULAR_PROTEIN_METABOLIC_PROCESS | Genes annotated by the GO term GO:0032270. Any process that activates or increases the frequency, rate or extent of the chemical reactions and pathways involving a protein, occurring at the level of an individual cell. | 0.26 |
| REACTOME_INNATE_IMMUNITY_SIGNALING | Genes involved in Innate Immunity Signaling | 0.23 |
| POSITIVE_REGULATION_OF_SIGNAL_TRANSDUCTION | Genes annotated by the GO term GO:0009967. Any process that activates or increases the frequency, rate or extent of signal transduction. | 0.22 |
| INFLAMMATORY_RESPONSE | Genes annotated by the GO term GO:0006954. The immediate defensive reaction (by vertebrate tissue) to infection or injury caused by chemical or physical agents. The process is characterized by local vasodilation, extravasation of plasma into intercellular spaces and accumulation of white blood cells and macrophages. | 0.22 |
| KEGG_T_CELL_RECEPTOR_SIGNALING_PATHWAY | T cell receptor signaling pathway | 0.20 |
| KEGG_JAK_STAT_SIGNALING_PATHWAY | Jak-STAT signaling pathway | 0.19 |
| DEFENSE_RESPONSE | Genes annotated by the GO term GO:0006952. Reactions, triggered in response to the presence of a foreign body or the occurrence of an injury, which result in restriction of damage to the organism attacked or prevention/recovery from the infection caused by the attack. | 0.17 |
| IMMUNE_RESPONSE | Genes annotated by the GO term GO:0006955. Any immune system process that functions in the calibrated response of an organism to a potential internal or invasive threat. | 0.17 |
| KEGG_CYTOKINE_CYTOKINE_RECEPTOR_INTERACTION | Cytokine-cytokine receptor interaction | 0.16 |
| PROTEIN_KINASE_CASCADE | Genes annotated by the GO term GO:0007243. A series of reactions, mediated by protein kinases, which occurs as a result of a single trigger reaction or compound. | 0.15 |
| RESPONSE_TO_WOUNDING | Genes annotated by the GO term GO:0009611. A change in state or activity of a cell or an organism (in terms of movement, secretion, enzyme production, gene expression, etc.) as a result of a stimulus indicating damage to the organism. | 0.15 |
| IMMUNE_SYSTEM_PROCESS | Genes annotated by the GO term GO:0002376. Any process involved in the development or functioning of the immune system, an organismal system for calibrated responses to potential internal or invasive threats. | 0.15 |
| REACTOME_SIGNALING_IN_IMMUNE_SYSTEM | Genes involved in Signaling in Immune system | 0.13 |
| REGULATION_OF_APOPTOSIS | Genes annotated by the GO term GO:0042981. Any process that modulates the occurrence or rate of cell death by apoptosis. | 0.11 |
| REGULATION_OF_PROGRAMMED_CELL_DEATH | Genes annotated by the GO term GO:0043067. Any process that modulates the frequency, rate or extent of programmed cell death, cell death resulting from activation of endogenous cellular processes. | 0.11 |
| POSITIVE_REGULATION_OF_BIOLOGICAL_PROCESS | Genes annotated by the GO term GO:0048518. Any process that activates or increases the frequency, rate or extent of a biological process. Biological processes are regulated by many means; examples include the control of gene expression, protein modification or interaction with a protein or substrate molecule. | 0.11 |
| POSITIVE_REGULATION_OF_CELLULAR_PROCESS | Genes annotated by the GO term GO:0048522. Any process that activates or increases the frequency, rate or extent of cellular processes, those that are carried out at the cellular level, but are not necessarily restricted to a single cell. For example, cell communication occurs among more than one cell, but occurs at the cellular level. | 0.10 |
| APOPTOSIS_GO | Genes annotated by the GO term GO:0006915. A form of programmed cell death induced by external or internal signals that trigger the activity of proteolytic caspases, whose actions dismantle the cell and result in cell death. Apoptosis begins internally with condensation and subsequent fragmentation of the cell nucleus (blebbing) while the plasma membrane remains intact. Other characteristics of apoptosis include DNA fragmentation and the exposure of phosphatidyl serine on the cell surface. | 0.10 |
| PROGRAMMED_CELL_DEATH | Genes annotated by the GO term GO:0012501. Cell death resulting from activation of endogenous cellular processes. | 0.10 |
| INTRACELLULAR_SIGNALING_CASCADE | Genes annotated by the GO term GO:0007242. A series of reactions within the cell that occur as a result of a single trigger reaction or compound. | 0.08 |
| CELL_DEVELOPMENT | Genes annotated by the GO term GO:0048468. The process whose specific outcome is the progression of the cell over time, from its formation to the mature structure. Cell development does not include the steps involved in committing a cell to a specific fate. | 0.08 |
| SIGNAL_TRANSDUCTION | Genes annotated by the GO term GO:0007165. The cascade of processes by which a signal interacts with a receptor, causing a change in the level or activity of a second messenger or other downstream target, and ultimately effecting a change in the functioning of the cell. | 0.06 |
